# Correlation Analysis between Three-Dimensional Changes in Pharyngeal Airway Space and Skeletal Changes in Patients with Skeletal Class II Malocclusion following Orthognathic Surgery

**DOI:** 10.1155/2022/3995690

**Published:** 2022-01-11

**Authors:** Moonhwan Kim, Chung-Ju Hwang, Jung-Yul Cha, Sang-Hwy Lee, Young Joon Kim, Hyung-Seog Yu

**Affiliations:** ^1^Department of Orthodontics, Yonsei University College of Dentistry, 50-1 Yonsei-ro, Seodaemun-gu, Seoul 03722, Republic of Korea; ^2^Department of Orthodontics, Institute of Craniofacial Deformity, Yonsei University College of Dentistry, 50-1 Yonsei-ro, Seodaemun-gu, Seoul 03722, Republic of Korea; ^3^Department of Oral & Maxillofacial Surgery, Oral Science Research Center, Yonsei University College of Dentistry, 50-1 Yonsei-ro, Seodaemun-gu, Seoul 03722, Republic of Korea

## Abstract

**Introduction:**

Studies on the pharyngeal airway space (PAS) changes using three-dimensional computed tomography (CT) have shed more light on patients with Class III than Class II malocclusion. This paper focuses on analyzing the long-term changes in the PAS and evaluating the postoperative association between these PAS and skeletal changes in patients with skeletal Class II malocclusion who have undergone orthognathic surgery.

**Methods:**

The records of 21 patients with skeletal Class II malocclusion who had undergone orthognathic surgery were included. The anatomical modifications in both jaws, changes in volume, sectional area (SA), minimum sectional area (MSA), and anterior-posterior (AP) and transverse (TV) width in the airway at one month before surgery (*T*0), and one month (*T*1) and one year (*T*2) after surgery were analyzed using CT images. The association between the skeletal and airway changes was evaluated between *T*0, *T*1, and *T*2.

**Results:**

After surgery, the ANS, A point, and PNS demonstrated significant posterior and superior movement. The B point and the pogonion exhibited substantial anterior and superior movement. The total and inferior oropharyngeal volumes (vol 3, vol 4) notably increased, while the nasopharyngeal volume (vol 1) decreased. The anterior-posterior movement at the ANS and PNS after surgery was significantly associated with the total volume, vol 2, vol 3, SA 1, MSA, and TV width 1, while substantial association with the total volume was found at the pogonion.

**Conclusion:**

Thus, an ideal treatment plan can be formulated for patients with skeletal Class II malocclusion by considering the postoperative PAS changes.

## 1. Introduction

Skeletal Class II malocclusion involves an anteroposterior difference in the maxillary and mandibular bones, which is followed by a vertical disparity [1]. On an average, approximately 15.5% of the individuals examined had Class II malocclusion, 50.1% had Class I, and 2.7% had Class III [2]. On the other hand, the prevalence and treatment demand of Class III malocclusion (16.7% and 38.1%, respectively) were higher than that of Class II malocclusion (13.1% and 28.6%, respectively) in the Korean population [3, 4].

Orthognathic surgery is a treatment option for the improvement of facial aesthetics and establishment of ideal occlusion in patients with severe skeletal discrepancies [5]. Following orthognathic surgery, the pharyngeal airway space (PAS) goes through substantial changes owing to its functional and anatomical relationships with both the maxilla and mandible [6].

Studies conducted in the past focused mainly on the connection between orthognathic surgery and PAS using two-dimension (2D) lateral cephalometric analysis [7]. However, despite its effectiveness in computing the size of the airways in the sagittal plane, it lacked accuracy in depicting the anatomy in three dimensions. Moreover, the most crucial information lies in the axial images perpendicular to the airflow pathway, and lateral cephalometric images fail to illustrate this [8]. On the other hand, computed tomographic data reveals precise images of the airway in 3D, including the axial plane. Thus, the use of computed tomographic data for three-dimensional (3D) reconstructions is recommended for the airway change analysis following surgery [8].

Previous studies have largely shed light on the changes in PAS following orthognathic surgeries for patients with Class III with corrections in either one jaw or both jaws [9, 10]. Moreover, literature reviews revealed the scarcity of studies dedicated to disclosing the relationship between the two in patients with Class II malocclusion [11]. These studies are lacking in the Asian population, especially because a much lower proportion of Asian patients with Class II malocclusion undergo orthognathic surgery compared to those with Class III malocclusion, resulting in difficulty in long-term data accumulation for such a population [12].

This study is aimed at measuring the long-term changes and evaluate the association between postoperative skeletal changes and PAS changes, concerning aspects such as the airway volume, cross-sectional area, and anterior-posterior (AP) and transverse (TV) width, using 3D multidetector computed tomography (MDCT) in patients with Class II malocclusion who underwent bimaxillary orthognathic surgeries.

## 2. Materials and Methods

### 2.1. Subjects

The study sample was retrospectively established using the records of 21 patients (6 males, 15 females; mean age, 29.82 ± 5.66 years) with skeletal Class II malocclusion, who had undergone surgeries such as Le Fort I osteotomy without maxillary surgical expansion, mandibular advancement by bilateral sagittal split ramus osteotomy (BSSRO), advanced genioplasty, and rigid fixation using plate and screw. The Yonsei Dental Hospital institutional review board (CRNo: 2-2021-0021) approved this study, and informed consent agreements were signed by the participants. All operations were performed by the same surgeon. Patients with craniofacial anomalies, severe facial asymmetry, surgical maxillary expansion, history of adenoidectomy or tonsillectomy, pathologic pharyngeal symptoms, and a body mass index (BMI) greater than 25 kg/m^2^ were excluded. Patients underwent orthodontic treatments before the surgeries; mandibular premolar extractions in ten cases, bimaxillary premolar extraction in six cases, and no extraction in five cases. The characteristics of the samples are described in Table 1 (ANB > 5°, Wits > 3.0 mm, body mass index (BMI) in the normal range). The normal value and standard deviation in the cephalometric analyses were based on the results of the cephalometric analysis study of Korean adults with normal occlusion that was conducted by Korean Association of Orthodontists (KAO).

### 2.2. Methods

#### 2.2.1. Three-Dimensional (3D) Reconstruction Using CT Scanning

**Figure 1:**
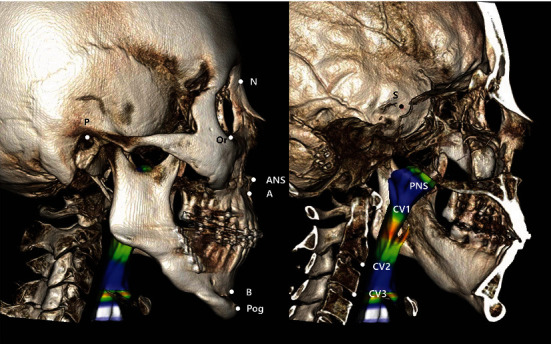
Landmarks. N: Nasion; Or: Orbitale; ANS: anterior nasal spine; A: A point; PNS: posterior nasal spine; B: B point; Pog: pogonion; S: Sella; P: Porion; CV 1: the most anteroinferior point of the first cervical vertebrae; CV 2: the most anteroinferior point of the second cervical vertebrae; CV 3: the most anteroinferior point of the third cervical vertebrae.

**Figure 2:**
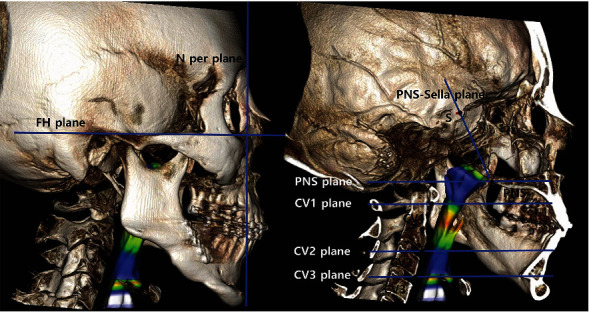
Reference planes. FH plane: the plane constructed by the right and left Porion and right Orbitale; N perpendicular plane: the vertical plane perpendicular to the FH plane and midsagittal plane passing through Nasion; PNS-Sella plane: the plane perpendicular to the midsagittal plane, passing through PNS and Sella; PNS plane: the plane parallel to the FH plane and perpendicular to the midsagittal plane passing through PNS; CV1, 2, 3 plane: the plane parallel to the FH plane and perpendicular to the midsagittal plane passing through each CV1, 2, 3 in each.

Every patient underwent examinations one month before the surgery (*T*0) and one month after (*T*1) and one year after (*T*2) the surgery. For the scans, a spiral CT scanner (High Speed Advantage®, GE Medical System, Milwaukee, WI, USA) was used. The digital imaging and communications in medicine (DICOM) files were imported and reconstructed into 3D images using the Invivo Dental 6 program (Anatomage Inc., San Jose, CA, USA), and the images were reoriented using the midsagittal plane and the Frankfort horizontal (FH) plane. The FH plane was constructed on both sides of Porion and Rt. Orbitale. The midsagittal plane was drawn perpendicular to the FH plane passing through Nasion and Sella. The landmarks and reference planes used in this study are listed in Figures 1 and 2. For uniform consistency, the values of the threshold were modified to -604.3 and -1000 HU (Houndsfield units); these were applied to preclude the possible interferences of soft and hard tissues in the images [17].

#### 2.2.2. Measurement of the Skeletal and Airway Changes (Volume, Sectional Area (SA), Minimum Sectional Area (MSA), Anterior-Posterior (AP), and Transverse (TV) Width)

**Figure 3:**
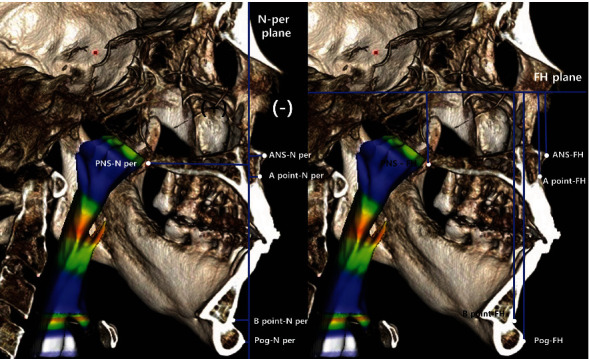
The skeletal measurement of the maxilla and mandible. ANS-N per: the linear distance between N-perpendicular plane and ANS; ANS-FH: the linear distance between FH plane and ANS; A point-N per: the linear distance between N-perpendicular plane and A point; A point-FH: the linear distance between FH plane and A point; PNS-N per: the linear distance between N-perpendicular plane and PNS; PNS-FH: the linear distance between FH plane and PNS; B Point-N per: the linear distance between N-perpendicular plane and B point; B point-FH, the linear distance between FH plane and B point; Pog-N per: the linear distance between N-perpendicular plane and pogonion; Pog-FH: the linear distance between FH plane and pogonion.

The FH plane was set as the horizontal reference, and the N-perpendicular plane was used as the vertical reference plane. The method for measurement of the skeletal changes of the maxilla (ANS, PNS, A point) and mandible (B point, pogonion) is described in Figure 3. The N-perpendicular plane was constructed perpendicular to the FH plane and midsagittal plane passing through the Nasion.

In order to analyze the skeletal changes, the distances between the reference points of the maxilla (ANS, PNS, A point), mandible (B point, pogonion), and reference plane (FH plane, N perpendicular plane) were measured at *T*0, *T*1, and *T*2. The maxillary and mandibular reference points were positive when they were located posterior to the N perpendicular plane (Figure 3).

**Figure 4:**
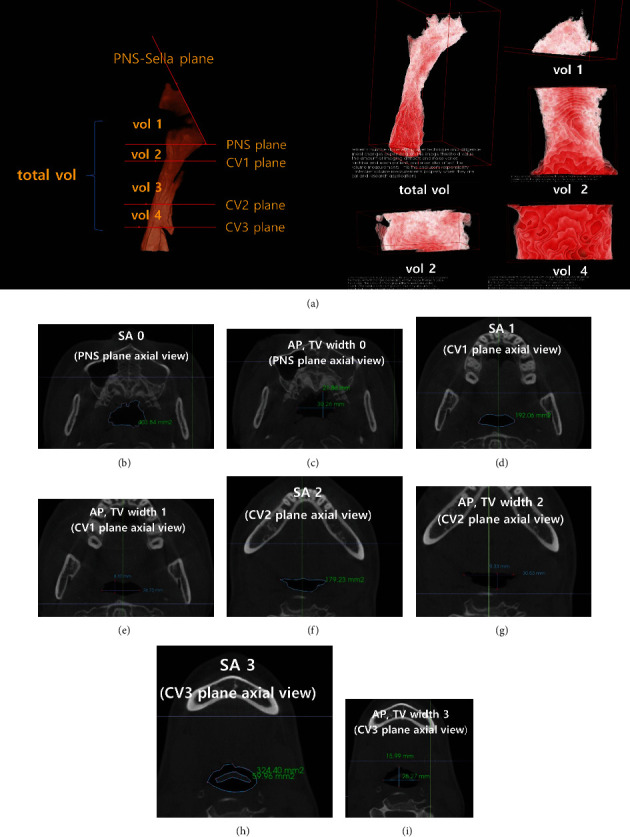
Measurement of the volume, SA, AP, and TV width measurement of the airway. (a) The subdivision of the pharyngeal airway space volume. (b) SA 0: the sectional area in the axial view of the PNS planes. (c) AP, TV width 0: AP, TV width in the axial view of the PNS planes. (d) SA 1: the sectional area in the axial view of the CV1 plane. (e) AP, TV width 1: AP, TV width in the axial view of the CV1 plane. (f) SA 2: the sectional area in the axial view of the CV2 plane. (g) AP, TV width 2: AP, TV width in the axial view of the CV2 plane. (h) SA 3: the sectional area in the axial view of the CV3 plane. (i) AP, TV width 3: AP, TV width in the axial view of the CV3 plane.

For the volumetric analysis of the PAS, the total volume of the pharynx, which consists of the nasopharynx and oropharynx, was assessed. The method and its reference planes that subdivide the pharynx are described in Figures 2 and 4(a). The methods for the measurement method of the airway changes (volume, sectional area (SA), minimum sectional area (MSA), anterior-posterior (AP), and transverse (TV) width) is described in Figure 4. The nasopharyngeal volume (vol 1) is the airway space that has bordered by the Sella-PNS and PNS plane. The Sella-PNS plane was constructed perpendicular to the midsagittal plane passing through the PNS and Sella. The PNS plane was constructed parallel to the FH plane and perpendicular to the midsagittal plane passing through PNS. Moreover, the oropharyngeal volume is the space between the PNS and CV 3 planes. The oropharyngeal volume was divided into volumes 2, 3, and 4 by the CV 1 and CV 2 planes (Figures 2 and 4(a)). The CV1, CV2, and CV3 planes were constructed parallel to the FH plane and perpendicular to the midsagittal plane passing through the CV1, CV2 and CV3, respectively.

The SA and AP and TV width in the axial view of the PNS, CV1, CV2, and CV3 planes were measured. (Figures 4(b)–4(i)) MSA was measured automatically using the airway module of the Invivo Dental 6 program between the PNS and CV3 planes.

### 2.3. Statistical Analysis

In order to test the intraexaminer reproducibility, seven randomly selected images were repeatedly measured by the same examiner within a minimum of two weeks following the first measurements, and the two measurements were compared by applying the paired *t*-test and Spearman's correlation coefficients. The Shapiro-Wilk test was considered when calculating the normal distribution. *P* value less than 0.05 signified statistical significance and for statistical analyses were performed using SPSS version 21.0 (SPSS Inc., Illinois, USA).

## 3. Results

1. The intraexaminer reliability

The intraexaminer reliability test revealed no notable contrast (*p* > 0.05). Intraclass correlation coefficients were found to be higher than 0.91 (mean of 0.93, with range of 0.91-0.94).

**Figure 5:**
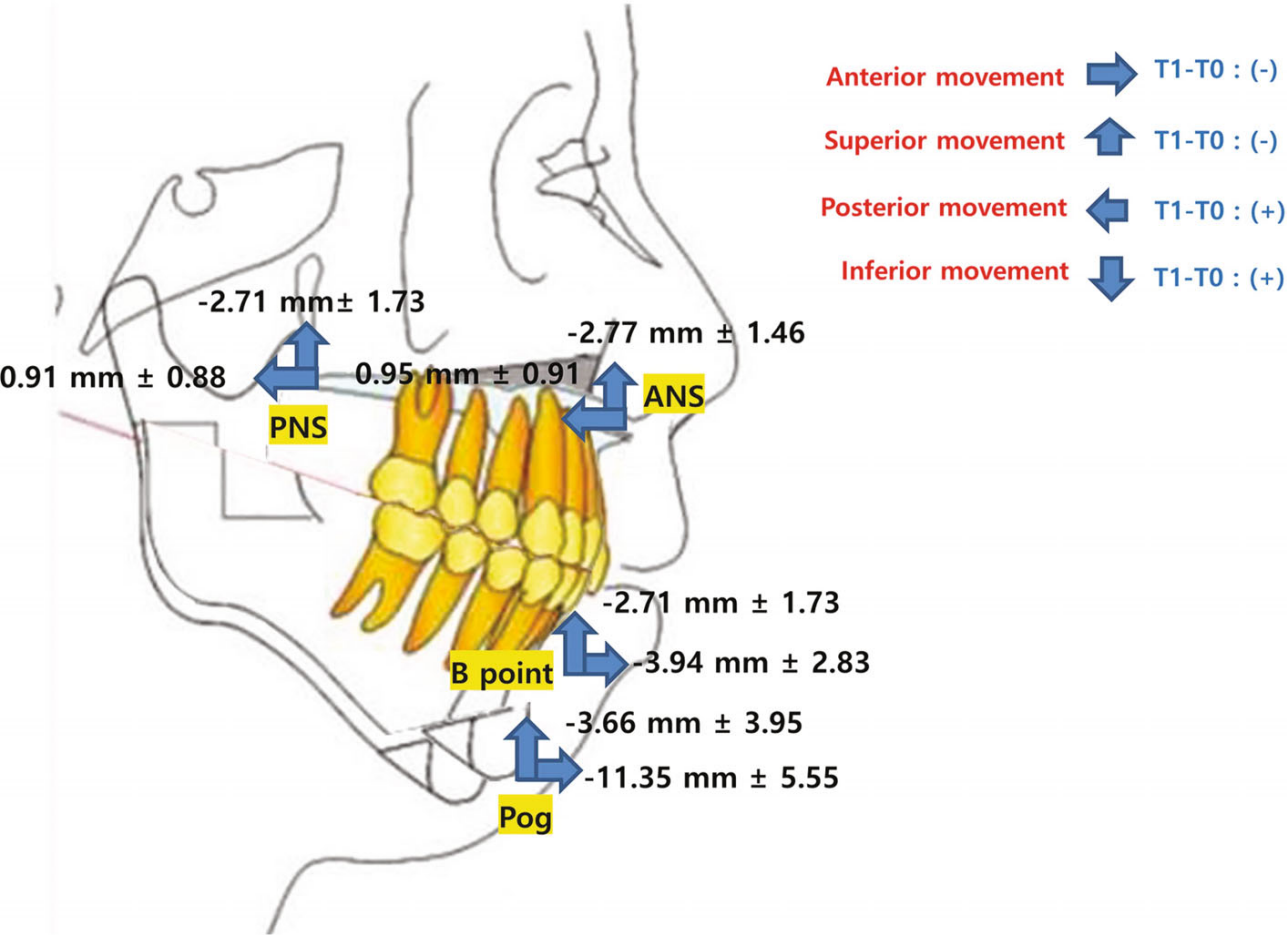
The summary of the skeletal changes of maxilla and mandible between *T*1 and *T*0.

(2) Anterior-posterior, vertical skeletal changes in the maxilla and mandible (*T*0-*T*1-*T*2, ANOVA test, Table 2 and Figure 5)

The summarized results of the anterior-posterior, vertical skeletal changes in the maxilla, mandible following AVONA between *T*0, *T*1, and *T*2 values are described in Table 2. The maxilla moved significantly posteriorly by an average of 0.954, 0.986, and 0.913 mm at the ANS, A point, and PNS, respectively, following surgery (*T*1-*T*0). The maxilla at the ANS, A point, and PNS moved significantly superiorly by an average of 2.777, 2.710, and 2.716 mm, respectively. The mandible moved significantly anteriorly by an average of 3.942 mm and 11.353 mm at B point and pogonion, respectively, and superiorly by an average of 1.480 mm and 3.670 mm at B point and pogonion, respectively. At the one-year follow-up (*T*2-*T*1), no notable positional variation of the maxilla was detected; however, the mandible moved to a significantly posterior position by an average of 0.178 mm at point B and 0.135 mm at pogonion. Moreover, an inferior relocation of 0.130 mm was noted at point B. The summary of the average skeletal changes in the maxilla and mandible between *T*1 and *T*0 in the 21 samples is described in Figure 5.

The mean and standard deviation of skeletal change at ANS, PNS, B point, and pogonion after surgery are obtained.

**Figure 6:**
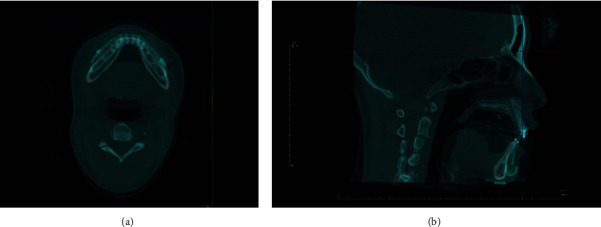
The superimposition of the PAS between *T*1 and *T*0 in the 3D CT image. (a) The superimposition in the axial view between *T*1 and *T*0. (b) The superimposition in the sagittal view between *T*1 and *T*0.

(3) The volume (mm^3^), SA, MSA (mm^2^), and AP TV width (mm) changes of the airway (*T*0-*T*1-*T*2, ANOVA test, Table 3, and Figure 6)

The summarized results of the volume (mm^3^), SA, MSA (mm^2^), and AP TV widths (mm) changes of the airway after AVONA analysis between *T*0, *T*1, and *T*2 are described in Table 3. The total and oropharyngeal volume (vol 3, vol 4) significantly increased, while the nasopharyngeal volume (vol 1) substantially decreased following surgery (*T*1-*T*0). At the one-year follow-up (*T*2-*T*1), no crucial changes in the airway volume were noted.

SA 2, SA 3, and MSA significantly increased following surgery (*T*1-*T*0). At the one-year follow-up (*T*2-*T*1), no crucial changes in SA and MSA were noted.

AP width 1, 2, and 3 significantly increased following surgery (*T*1-*T*0). At the one-year follow-up (*T*2-*T*1), no crucial transformations in AP and TV width were noted.

The superimposition image of PAS between *T*1 and *T*0 in 3D CT image in the axial and sagittal planes in one sample, which illustrates the change close to mean airway change among the 21 samples is described in Figure 6.

(4) Multiple linear regression analysis was done for volume, SA, and AP and TV width changes due to skeletal changes (at ANS, PNS, B point, and pogonion) between *T*1 and *T*0. (Tables 4 and 5)

The multiple linear regression analysis results of the volumetric changes due to skeletal changes (at PNS, ANS, B point, and pogonion) between *T*1 and *T*0 are described in Table 4. The anterior-posterior movement at the ANS following surgery (*T*1-*T*0) was significantly associated with the change in the total vol (*β* = −0.552, *p* = 0.013), vol 2 (*β* = −0.479, *p* = 0.036), and vol 3 (*β* = −0.481, *p* = 0.035). The vertical movement at the ANS following surgery (*T*1-*T*0) was significantly associated with a change in the vol 4 (*β* = −0.490, *p* = 0.024). The anterior-posterior movement at the PNS following surgery (*T*1-*T*0) was significantly associated with the change in the total vol (*β* = −0.577, *p* = 0.020), vol 2 (*β* = −0.522, *p* = 0.036), vol 3 (*β* = −0.534, *p* = 0.034), and vol 4 (*β* = −0.547, *p* = 0.025). The anterior-posterior movement at the pogonion following surgery (*T*1-*T*0) was significantly associated with the change in the total vol (*β* = −0.481, *p* = 0.043) (Table 4).

The multiple linear regression analysis results for the sectional area (SA), minimum sectional area (MSA), and AP and TV width changes caused by the skeletal changes at the PNS and ANS between *T*1 and *T*0 are described in Table 5. The anterior-posterior movement at the ANS and PNS following surgery (*T*1-*T*0) was significantly associated with changes in SA 1 (*β* = −0.485, *p* = 0.025; *β* = −0.541, *p* = 0.024, respectively), MSA (*β* = −0.462, *p* = 0.032; *β* = −0.618, *p* = 0.009, respectively), and TV width 1 (*β* = −0.454, *p* = 0.042; *β* = −0.489, *p* = 0.048, respectively). The vertical movement at the PNS following surgery (*T*1-*T*0) was significantly associated with the change in MSA (*β* = −0.525, *p* = 0.022). The anterior-posterior movement at the PNS after surgery (*T*1-*T*0) was significantly associated with the change in AP width 2 (*β* = −0.549, *p* = 0.028) (Table 5). The anterior-posterior and vertical change at B point and pogonion following surgery (*T*1-*T*0) were insufficiently related to the changes in SA, MSA, and AP and TV width.

(5) Multiple linear regression analysis was conducted for volume, SA, and AP and TV width changes owing to skeletal changes (at ANS, PNS, B point, and pogonion) between *T*2 and *T*1. (Tables 6 and 7)

The multiple linear regression analysis results of the volumetric changes caused by the skeletal changes (at PNS, ANS, B point, and pogonion) between *T*2 and *T*1 are described in Table 6. The anterior-posterior and vertical change at the ANS and PNS between *T*2 and *T*1 were insufficiently related to the changes in airway volume (Table 6). The anterior-posterior and vertical changes at B point and pogonion between *T*2 and *T*1 were also insufficiently related to the changes in the airway volume (Table 6).

The multiple linear regression analysis results of sectional area (SA), minimum sectional area (MSA), AP, TV width changes by skeletal changes at PNS, ANS between *T*2 and *T*1 are described in Table 7. The anterior-posterior and vertical changes at PNS, ANS between *T*2 and *T*1 were insufficiently related to the changes in the SA, MSA, and AP and TV width (Table 7). The anterior-posterior and vertical changes at B point and pogonion between *T*2 and *T*1 were also insufficiently related to the changes in the SA, MSA, and AP and TV width.

(6) The airway changes in the volume, SA, and AP and TV width following surgery (between *T*0 and *T*0) according to the extraction and non-extraction of the premolars in the presurgical orthodontic treatment phases (Table 8)

Patients had undergone orthodontic treatments with mandibular premolar extractions in ten cases, bimaxillary premolar extraction in six cases, and no extraction in five cases. The Mann–Whitney *U* test was used for the comparison of the airway change following surgery between the premolar extraction group (*n* = 16) and nonextraction (*n* = 5) group. There was no significant difference between the extraction and non-extraction group regarding the change in the airway (volume, SA, MSA, AP width, and TV width) following surgery (between *T*1 and *T*0) (Table 8).

The Kruskal-Wallis test was used for the comparison of the airway change following surgery between the bimaxillary premolar extraction (*n* = 6), mandibular premolar extraction (*n* = 10), and nonextraction (*n* = 5) groups. There was no significant difference in the changes in the airway (Volume, SA, MSA, AP width, and TV width) between the three groups following surgery (between *T*1 and *T*0).

## 4. Discussion

In this study, we analyzed the amount of three-dimensional (3D) movement of the maxilla and mandible following orthognathic surgery. The three-dimensional (3D) movement of the maxilla and mandible following orthognathic surgery is categorized by anterior-posterior, vertical, and transverse movement. Horizontally, the FH plane was set as a reference, and vertically, the N-perpendicular plane was used. The anterior-posterior and vertical changes of maxilla (ANS, PNS, A point) and mandible (B point, pogonion) were analyzed between *T*0, *T*1, and *T*2 (Figure 3). The transverse movement can be evaluated using the midsagittal plane; however, in this study, the transverse movement was not evaluated because patients with facial asymmetry and maxillary surgical expansion were excluded. The maxilla moved significantly posteriorly by an average of 0.954, 0.986, and 0.913 mm at the ANS, A point, and PNS, respectively, and significantly superiorly by an average of 2.777, 2.710, and 2.716 mm, respectively, following surgery (*T*1-*T*0). The mandible moved significantly anterior by an average of 3.942 and 11.353 mm at B point and pogonion, respectively, and superior by an average of 1.480 and 3.670 mm at B point and pogonion, respectively (Table 2, Figure 5).

In orthognathic surgery, PAS must be taken into consideration because the airway's capacity undergoes substantial changes owing maxillary and mandibular movements. Such changes have been verified in other studies, which confirmed the relationship between the protrusion of both the jaws and the increase in the volume of the PAS [18, 19]. In general, a maxillomandibular advancement (MMA) of 10 mm on average for patients with obstructive sleep apnea (OSA) results in a mean increase of 4.75 mm (ranging from 3.15 to 6.35) in the PAS, and an average expansion of 7.35 cm^3^ (ranging from 5.35 to 9.34) in the pharyngeal airway volume (PAV) in average [20]. On the other hand, our study mainly concentrated on the PAS change in Korean patients with skeletal Class II patients who did not have OSA and undergone orthognathic surgeries. The results demonstrated a notable increase in the total volume, which consisted of a significant increase in the inferior oropharyngeal volume (vol 3, vol 4), and a significant decrease in the nasopharyngeal volume (vol 1) in the 21 patients who underwent mandibular advancement with superior and posterior movement of the maxilla. The decrease in the nasopharyngeal volume (vol 1) following surgery is most likely due to the posterior movement of the maxilla; however, the reduction of the nasopharyngeal volume was compensated by the increase in the oropharyngeal volume (vol 3, vol 4) by mandibular advancement through BSSRO and advanced genioplasty. In conclusion, the total volume was increased despite a decrease in the nasopharyngeal volume, and the increased total volume after surgery was retained until one-year post-surgery (Table 3).

The changes in the PAS caused by orthognathic surgery play a very important role in quality of the life of patients. Therefore, there are many studies on the correlation between orthognathic surgeries and the PAS change. In this study, we focused on the association between the skeletal changes and the long-term changes in the PAS, in aspects such as the airway volume, cross-sectional area, and anterior-posterior (AP) and transverse (TV) width, using 3D multidetector computed tomography (MDCT) in patients with skeletal Class II malocclusion who had bimaxillary orthognathic surgeries. The association between the skeletal and airway changes was tested between *T*0, *T*1, and *T*2 using multiple linear regression analysis (Tables 4–7). Our results support the findings of other investigations which suggest that the protrusion of the maxilla and mandible results in an increase of the PAS [21]. In this study, the anterior-posterior movement at the ANS following surgery (T1-T0) was significantly associated with the changes in the total vol, vol 2, and vol 3. The anterior-posterior movement at the PNS following surgery (*T*1-*T*0) was substantially related with the change in total vol, vol 2, vol 3, and vol 4. The positive value in the skeletal changes of the maxilla, mandible (*ΔT*1-*T*0 and *ΔT*2-*T*1) means posterior or inferior movement of maxilla or mandible. The negative value means the anterior or superior movement of maxilla or mandible. Jakobsone et al. [22] reports that 2.0 mm or more anterior movement of the maxilla significantly increases the nasopharyngeal airway volume. The anterior-posterior movement of the ANS and PNS following surgery (*T*1-*T*0) was significantly associated with the changes in SA 1, MSA, and TV width 1. This coincided with the results established by Burruneto et al. [23], who found a powerful and positive link between maxillary displacement and the MSA. The maxillary anterior movement could directly result in an increase in the nasopharyngeal space, which is related the posterior area of the maxilla. In addition, maxillary anterior movement results in mandibular autorotation. This results in the superior and anterior positioning of the tongue, hyoid bone, and genioglossus muscle. We assume that the maxillary anterior movement induces the increase in the PAS by these two mechanisms. The anterior-posterior movement at the pogonion following surgery (*T*1-*T*0) was notably related to the change in total volume. The mandibular anterior movement causes an anterior movement of the numerous soft tissues inserted at the mandible which results in an increase in the posterior airway space. Hart et al. [17] also reported that horizontal motions of the D-point had a substantial correlation with the increase in the oropharynx volumes as well as total airway. In this study, the vertical movement of the PNS following surgery (*T*1-*T*0) was significantly associated with a change in vol 4. The oropharyngeal volume (vol 4) can be increased by the superior movement of the maxilla, which causes mandibular autorotation, and the consequent anterior-superior positioning of the tongue, hyoid bone, and genioglossus muscle. This also concurred with outcomes observed by Hart et al. [17]. The significant association between the skeletal and airway changes was only observed between *T*1 and *T*0. The anterior-posterior and vertical change at ANS, PNS, B point, and pogonion between *T*2 and *T*1 were insufficiently related to the changes in the airway volume, SA, MSA, AP width, and TV width. We assume this result can be attributed to the minimal skeletal changes in the maxilla and mandible between *T*1 and *T*2. At one-year follow-up (*T*2-*T*1), no considerable positional variation of the maxilla was noted. Although the mandible moved to a significantly posterior position by an average of 0.178 mm at point B and 0.135 mm at the pogonion and an inferior relocation of 0.130 mm was noted at point B, the amount of skeletal change in the mandible between T2 and T1 was very small.

A significant increase in the PAS following the advancement of the maxilla and mandible with genioplasty conducted during orthognathic surgery was confirmed by a recent meta- analysis study [24]. In this study, all the participants underwent mandibular advancement using BSSRO and advanced genioplasty. The average anterior movement at B point was 3.94 mm compared to 11.35 mm at the pogonion. The anterior movement of the pogonion resulted from the advanced genioplasty and anterior motion of the mandible by BSSRO. The advancement of the pogonion was significantly associated with the change in total volume following surgery (*T*1-*T*0). Anterior movement of the chin is associated with advancement of the genial tubercles, and combined with the corresponding movement of the hyoid, is likely to result in greater flow to the airway [25].

The smallest cross-sectional area is especially important in OSA. The smallest cross-sectional area of the airway in adults with sleep apnea is approximately 40-67.1 mm^2^ [26–30]; for reference, the average upper airway size is approximately 141.9-149.3 mm^2^ in adults without OSA [29, 31]. In this study, the mean MSA at T0 was 101.98 mm^2^, which is larger than the 40-67.1 mm^2^ in patients with OSA but smaller than 141.9-149.3 mm^2^, the mean in normal adults. The mean MSA increased to 192.20 mm^2^ at T1 and dropped to 171.90 mm^2^ at T2. The PAS must be assessed in patients with retrognathic mandibles, while paying more attention paid to those with more severe malocclusion. When signs of snoring or OSA are detected, a sleep study that includes polysomnography could be favorable for patients with Class II malocclusion and significant mandibular retrognathism.

When diverse densities of Hounsfield units are utilized, CTs provides more advantageous illustration of the soft tissues and airway [32]. The radiation dose of CT depends on the type of equipment used and exposure parameters, particularly on the designated field of view. Latest CT equipment can remarkably reduce the doses by using low-dose protocols [33]. Although numerous arguments suggest that CT scans expose patients to more radiation than traditional digital films [34], the advantages are conspicuous for orthodontic patients who needs surgical intervention: extensive sagittal and lateral examinations, availability of various two-dimensional and three-dimensional (3D) computational simulations, and better understanding of the case when making templates for surgical guides, therefore allowing more foreseeable outcomes [35].

Regarding the accepted pattern of airway segmentation, a concurrence has not been established yet in published papers. Martínez et al. [36] proposed using bodily boundaries to merge superior respiratory tract and subregion analyses and allow comparisons in ensuing research. In this study, we cited the methodologies of Martínez et al. [36] and Hart et al. [17] for PAS analysis (the nasopharynx and oropharynx). It was difficult to determine the involvement of the airway area between the fourth and sixth cervical vertebrae in the CT images of all the samples. As a result, the analysis of the hypopharyngeal area was excluded from this study.

Furthermore, the patient's position and disposition of the head influenced the airway [37]. Every scan obtained in this study had the patient in the supine position with the FH plane, perpendicular to the floor. We believed that the supine position provided better anatomical detail than the upright position (which is used usually in come-beam CT) because it is a common sleeping pose with minimum bodily movements. Moreover, OSA mainly occurs in such position owing to the deformation of the superior pharyngeal tissue caused by gravity and relaxation of the muscle tone. However, the issue regarding supine position is in still under debate because it is very difficult to consider supine position in an awake participant comparable with a supine sleeping position. Therefore, we are unable to conclude that the result of this study conducted using the supine position, which is used usually in CT scan represents the airway change in the real sleeping position of patients at bedtime. Using cone beam computed tomography (CBCT) scanner, Holsbeke et al. [38] compared the airway in two positions: lying down and sitting up. In supine position, the airway became substantially smaller, and resistance increased. This can be explained by the fact that the weight vectors of the surrounding tissues are aligned perpendicular to the airway walls in this position, increasing the load on the airway muscles.

In this study, three-dimensional computed tomography (3DCT) scans were used for each patient. They were taken one month before surgery (*T*0), one month after surgery (*T*1), and one year after surgery (*T*2). Kawakami et al. [39] proposed that it is most appropriate to take images in the following month because it allows time for the healing of post-surgical swellings that constricts the airway. Furthermore, the preoperative orthodontic treatment with premolar extraction could affect the PAS [40]. Therefore, we also analyzed the significant differences in the airway change in the volume, SA, and AP and TV width following surgery (between *T*1 and *T*0) according to the extraction and non-extraction of premolars in presurgical orthodontic treatment. However, there was no significant difference between the extraction and nonextraction groups in the changes in the airway (volume, SA, AP width, TV width, and MSA) following surgery (between *T*1 and *T*0) (Table 8). In this study, presurgical orthodontic treatment was conducted in every participant, and each of them underwent a 3D CT examination one month before surgery when the presurgical orthodontic treatment was completed (premolar extraction space closure was achieved) and was not at the initial stage of presurgical orthodontic treatment. We assume this eliminates the influence of retracted anterior teeth and protracted posterior teeth owing to premolar extraction on the PAS.

A limitation of this study lies in it being a retrospective study. It examines the postoperative influences on the changes in the airway and provides quantified data, without qualitative information obtained directly from the patients. Even though the findings in this paper have been verified using mandibular advancement as a solid method for expansion of the PAS, the correlation between our conclusions and the patient's sleeping criterion can be assessed by utilizing polysomnography and questionnaire survey preoperatively and postoperatively (at *T*0 and *T*1). Another limitation is that we used cervical vertebrae to divide upper airway volume. Although many studies regarding the airway have been conducted using the vertebrae as a reference point for division of the airway, the position of the cervical vertebrae may vary between scans. Therefore, recently Carlo et al. [41] proposed an alternative strategy to characterize the upper airway in patients of orthognathic surgery. The other limitation was the small number of participants employed in this study. Owing to this drawback, traits could not be sorted by sex. Thus, to evaluate the postoperative modifications in the PAS more profoundly, long-term prospective studies on a larger sample size of patients with skeletal Class II malocclusion were warranted.

## 5. Conclusion

This study measured the long-term changes in the superior air tract of 21 patients with skeletal Class II malocclusion and who had undergone orthognathic surgery, using 3D CT. These measurements were used to evaluate the association between skeletal and airway modifications post-operatively. The results obtained from this study can be utilized as references by surgeons and orthodontists to establish an optimal treatment plan and consider the merits and demerits of orthognathic surgery concerning the PAS.

## Figures and Tables

**Table 1:** Characteristics of the sample (*N* = 21, male 15, female 6).

Sample		Mean	SD	Normal	SD
Age		29.82 years	5.66		
Steiner analysis [13]	SNA	79.19°	3.79	82.05	3.22
	SNB	70.30°	4.20	79.79	3.12
	ANB	8.88°	2.92	2.26	1.79
	Go-GN-SN	48.00°	8.56	31.80	5.53
Wits analysis [14]	Wits	3.47 mm	4.34	-2.49	2.48
Tweed analysis [15]	IMPA	95.30°	9.58	96.25	6.50
	FMA	37.87°	8.62	23.5	5.02
Body mass index [16]	BMI	20.54 kg/m^2^	2.54	24.5	3

SD: standard deviation; SNA: sella, nasion, A point; SNB: sella, nasion, B point: ANB: A point, nasion, B point; Go-GN-SN: gonion-gnathion-SN plane; IMPA: incisor mandibular plane angle; FMA: Frankfort mandibular plane angle; BMI: body mass index.

**Table 2:** Anterior-posterior, vertical skeletal changes in maxilla, mandible (*ΔT*1-*T*0 and *ΔT*2-*T*1).

	*ΔT*1-*T*0		*ΔT*2-*T*1	
	Mean	SE	Mean	SE
ANS-N per (mm)	0.954^∗∗∗^	0.200	0.029	0.124
ANS-FH (mm)	-2.777^∗∗∗^	0.319	0.024	0.143
A point-N per (mm)	0.986^∗∗∗^	0.211	0.053	0.128
A point-FH (mm)	-2.710^∗∗∗^	0.328	0.002	0.132
PNS-N per (mm)	0.913^∗∗∗^	0.193	0.023	0.127
PNS-FH (mm)	-2.716^∗∗∗^	0.378	0.070	0.162
B point-N per (mm)	-3.942^∗∗∗^	0.620	0.178^∗∗∗^	0.025
B point-FH (mm)	-1.480^∗∗^	0.457	0.130^∗∗^	0.043
Pog-N per (mm)	-11.353^∗∗∗^	1.212	0.135^∗∗^	0.038
Pog-FH (mm)	-3.670^∗∗∗^	0.863	0.106	0.064

Positive value means posterior or inferior movement of maxilla or mandible. Negative value means anterior or superior movement of maxilla or mandible. *T*0: one month before surgery; *T*1: one month after surgery; *T*2: one year after surgery. ^∗^*p* < 0.05, ^∗∗^*p* < 0.01, and ^∗∗∗^*p* < 0.001.

**Table 3:** The airway changes between *T*0, *T*1, and *T*2.

	*ΔT*1-*T*0		*ΔT*2-*T*1	
	Mean	SE	Mean	SE
Total vol (mm^3^)	3669.952^∗∗^	1253.148	214.619	1705.447
Vol 1 (mm^3^)	-924.524^∗∗^	290.506	140.238	242.652
Vol 2 (mm^3^)	213.381	377.480	367.381	471.678
Vol 1+vol 2 (mm^3^)	-711.143	566.608	507.619	652.248
Vol 3 (mm^3^)	2846.190^∗∗∗^	644.267	-285.571	934.736
Vol 4 (mm^3^)	1534.905^∗∗∗^	355.373	-7.429	358.278
SA 0 (mm^2^)	-14.038	24.443	40.145	22.070
SA 1 (mm^2^)	37.942	29.780	14.255	40.212
SA 2 (mm^2^)	128.490^∗∗∗^	22.124	-1.123	29.864
SA 3 (mm^2^)	44.629^∗^	20.249	25.466	19.964
MSA (mm^2^)	90.224^∗∗∗^	19.293	-20.300	20.037
AP width 0 (mm)	1.208	0.733	0.475	0.415
TV width 0 (mm)	-0.986	0.945	0.365	0.941
AP width1 (mm)	1.994^∗∗^	0.703	-0.510	0.732
TV width 1 (mm)	-0.473	1.231	1.129	1.680
AP width 2 (mm)	4.949^∗∗∗^	0.840	-0.766	1.025
TV width 2 (mm)	1.924	0.995	0.003	0.625
AP width 3 (mm)	2.404^∗∗^	0.726	0.215	0.894
TV width 3 (mm)	-0.084	0.646	0.022	0.549

A positive value indicates an increase in airway volume, SA, MSA, and AP TV width. A negative value indicates a decrease in airway volume, SA, MSA, and AP and TV width. *T*0: one month before surgery; *T*1: one month after surgery; *T*2: one year after surgery. ^∗^*p* < 0.05, ^∗∗^*p* < 0.01, and ^∗∗∗^*p* < 0.001.

**(a):** 

Maxilla	Total vol			Vol 1			Vol 2		
	*β*	SE	*p*	*β*	SE	*p*	*β*	SE	*p*
ANS-N per	-0.552	1259.795	0.013	-0.232	300.189	0.276	-0.479	398.438	0.036
ANS-FH	-0.157	787.961	0.445	0.412	187.759	0.061	-0.147	249.210	0.494
PNS-N per	-0.577	1466.564	0.020	0.040	329.204	0.856	-0.522	451.519	0.036
PNS-FH	-0.219	747.908	0.346	0.577	167.885	0.017	-0.367	230.262	0.129

**(b):** 

Maxilla	Vol 1+2			Vol 3			Vol 4		
	*β*	SE	*p*	*β*	SE	*p*	*β*	SE	*p*
ANS-N per	-0.438	599.314	0.053	-0.481	679.933	0.035	-0.375	352.899	0.075
ANS-FH	0.113	374.852	0.598	-0.134	425.276	0.532	-0.490	220.727	0.024
PNS-N per	-0.327	724.936	0.202	-0.534	774.404	0.034	-0.547	413.313	0.025
PNS-FH	0.051	369.698	0.838	-0.227	394.925	0.341	-0.441	210.779	0.065

**(c):** 

Mandible	Total vol			Vol 1			Vol 2		
	*β*	SE	*p*	*β*	SE	*p*	*β*	SE	*p*
B point-N per	-0.213	331.145	0.396	0.067	83.188	0.805	-0.076	105.259	0.773
B point-FH	0.037	764.267	0.896	-0.215	191.993	0.488	0.028	242.933	0.926
Pog-N per	-0.481	226.104	0.043	-0.354	56.800	0.154	-0.467	71.870	0.060
Pog-FH	-0.049	369.457	0.849	0.451	92.812	0.122	-0.153	117.437	0.577

**(d):** 

Mandible	Vol 1+2			Vol 3			Vol 4		
	*β*	SE	*p*	*β*	SE	*p*	*β*	SE	*p*
B point-N per	-0.016	159.516	0.951	-0.179	187.076	0.515	-0.401	95.056	0.124
B point-FH	-0.092	368.155	0.762	0.185	431.762	0.554	-0.059	219.384	0.838
Pog-N per	-0.493	108.917	0.050	-0.364	127.734	0.149	-0.252	64.903	0.271
Pog-FH	0.129	177.971	0.641	-0.190	208.720	0.507	-0.035	106.053	0.893

**(a):** 

Maxilla	SA 0			SA 1			SA 2		
	*β*	SE	*p*	*β*	SE	*p*	*β*	SE	*p*
ANS-N per	-0.312	27.065	0.175	-0.485	29.479	0.025	-0.381	23.670	0.092
ANS-FH	0.176	16.928	0.437	0.215	18.438	0.291	-0.322	14.805	0.149
PNS-N per	-0.316	31.997	0.226	-0.541	33.888	0.024	-0.473	27.300	0.062
PNS-FH	-0.080	16.317	0.755	0.029	17.282	0.896	-0.299	13.922	0.226

**(b):** 

Maxilla	SA 3			MSA		
	*β*	SE	*p*	*β*	SE	*p*
ANS-N per	-0.314	21.266	0.152	-0.462	19.265	0.032
ANS-FH	-0.428	13.301	0.056	-0.400	12.049	0.060
PNS-N per	-0.389	25.837	0.131	-0.618	21.012	0.009
PNS-FH	-0.268	13.176	0.290	-0.525	10.715	0.022

**(c):** 

Maxilla	AP width 0			TV width 0			AP width 1			**TV width 1**		
	*β*	SE	*p*	*β*	SE	*p*	*β*	SE	*p*	*β*	SE	*p*
ANS-N per	-0.238	0.791	0.284	-0.116	1.110	0.626	-0.331	0.771	0.148	-0.454	1.279	0.042
ANS-FH	0.325	0.495	0.149	0.147	0.694	0.539	0.181	0.483	0.419	0.142	0.800	0.503
PNS-N per	-0.151	0.942	0.551	-0.100	1.286	0.707	-0.340	0.861	0.168	-0.489	1.469	0.048
PNS-FH	0.246	0.481	0.334	-0.006	0.656	0.983	0.172	0.439	0.475	-0.001	0.749	0.997

**(d):** 

Maxilla	AP width 2			TV width 2			AP width 3			TV width 3		
	*β*	SE	*p*	*β*	SE	*p*	*β*	SE	*p*	*β*	SE	*p*
ANS-N per	-0.428	0.906	0.062	-0.321	1.080	0.157	-0.079	0.825	0.732	0.043	0.774	0.858
ANS-FH	-0.194	0.567	0.380	-0.344	0.676	0.130	-0.324	0.516	0.171	0.014	0.484	0.954
PNS-N per	-0.549	1.000	0.028	-0.423	1.240	0.096	-0.209	0.947	0.418	0.095	0.879	0.721
PNS-FH	-0.213	0.510	0.366	-0.355	0.632	0.158	-0.329	0.483	0.208	0.092	0.449	0.730

**(a):** 

Maxilla	Total vol			Vol 1			Vol 2		
	*β*	SE	*p*	*β*	SE	*p*	*β*	SE	*p*
ANS-N per	0.006	0.025	0.980	0.059	0.307	0.762	-0.099	-0.441	0.664
ANS-FH	-0.125	-0.545	0.592	-0.538	-2.780	0.051	-0.225	-1.000	0.330
PNS-N per	0.008	0.033	0.974	0.062	0.297	0.769	-0.087	-0.387	0.703
PNS-FH	-0.161	-0.700	0.492	-0.433	-2.085	0.051	-0.264	-1.178	0.253

**(b):** 

Maxilla	Vol 1+2			Vol 3			Vol 4		
	*β*	SE	*p*	*β*	SE	*p*	*β*	SE	*p*
ANS-N per	-0.050	-0.230	0.820	-0.021	-0.089	0.930	0.172	0.754	0.460
ANS-FH	-0.363	-1.680	0.109	0.004	0.017	0.987	0.055	0.241	0.812
PNS-N per	-0.040	-0.183	0.857	0.007	0.028	0.978	0.091	0.395	0.697
PNS-FH	-0.352	-1.616	0.122	-0.029	-0.126	0.901	-0.047	-0.204	0.841

**(c):** 

Manbible	Total vol			Vol 1			Vol 2		
	*β*	SE	*p*	*β*	SE	*p*	*β*	SE	*p*
B point-N per	-0.454	-1.53	0.144	-0.262	-0.854	0.405	-0.406	-1.344	0.197
B point-FH	0.623	1.28	0.218	0.552	1.100	0.287	0.414	0.835	0.416
Pog-N per	0.302	0.927	0.367	0.196	0.584	0.567	0.112	0.337	0.741
Pog-FH	-0.809	-1.479	0.158	-0.468	-0.829	0.419	-0.492	-0.882	0.390

**(d):** 

Mandible	Vol 1+2			Vol 3			Vol 4		
	*β*	SE	*p*	*β*	SE	*p*	*β*	SE	*p*
B point-N per	0-.391	-1.290	0.214	-0.484	-1.643	0.119	-0.187	-0.613	0.548
B point-FH	0.505	1.015	0.324	0.630	1.304	0.210	0.405	0.812	0.428
Pog-N per	0.154	0.462	0.650	0.355	1.100	0.287	0.229	0.687	0.501
Pog-FH	-0.530	-0.948	0.357	-0.822	-1.514	0.148	-0.743	-1.323	0.203

**(a):** 

Maxilla	SA 0			SA 1			SA 2		
	*β*	SE	*p*	*β*	SE	*p*	*β*	SE	*p*
ANS-N per	0.129	0.585	0.566	0.125	0.544	0.593	0.139	0.608	0.550
ANS-FH	-0.257	-1.167	0.258	0.006	0.026	0.980	0.080	0.351	0.729
PNS-N per	0.153	0.712	0.485	0.154	0.676	0.507	0.084	0.362	0.721
PNS-FH	-0.326	-1.516	0.146	-0.092	-0.405	0.690	-0.019	-0.081	0.936

**(b):** 

Maxilla	SA 3			MSA		
	*β*	SE	*p*	*β*	SE	*p*
ANS-N per	0.342	1.576	0.132	0.197	0.869	0.395
ANS-FH	0.129	0.595	0.559	0.101	0.445	0.661
PNS-N per	0.303	1.366	0.188	0.243	1.075	0.296
PNS-FH	0.083	0.374	0.713	0.028	0.124	0.903

**(c):** 

Maxilla	AP width 0			TV width 0			AP width 1			TV width 1		
	*β*	SE	*p*	*β*	SE	*p*	*β*	SE	*p*	*β*	SE	*p*
ANS-N per	0.217	0.973	0.343	0.261	1.242	0.229	0.006	0.027	0.979	0.092	0.399	0.694
ANS-FH	-0.146	-0.655	0.520	-0.291	-1.381	0.183	0.063	0.272	0.789	-0.034	-0.149	0.883
PNS-N per	0.225	0.994	0.333	0.338	1.614	0.123	0.044	0.189	0.852	0.111	0.484	0.634
PNS-FH	-0.026	-0.115	0.909	-0.228	-1.091	0.289	-0.047	-0.204	0.841	-0.119	-0.519	0.610

**(d):** 

Maxilla	AP width 2			TV width 2			AP width 3			TV width 3		
	*β*	SE	*p*	*β*	SE	*p*	*β*	SE	*p*	*β*	SE	*p*
ANS-N per	0.155	0.682	0.503	0.071	0.312	0.759	0.413	2.062	0.053	0.219	0.969	0.345
ANS-FH	0.134	0.588	0.563	-0.146	-0.639	0.530	0.343	1.711	0.103	0.087	0.386	0.704
PNS-N per	0.125	0.542	0.594	-0.023	-0.102	0.920	0.301	1.366	0.188	0.248	1.109	0.281
PNS-FH	0.069	0.300	0.767	-0.172	-0.751	0.462	0.151	0.686	0.501	0.151	0.674	0.509

**Table 8:** Airway change (*ΔT*1-*T*0) comparison following surgery (*T*1-*T*0) between the premolar extraction and nonextraction groups (Mann–Whitney *U* test).

	Group	*N*	Mean (*ΔT*1-*T*0)	SD	*U*	*p*
Total vol (mm^3^)	Ext.	16	4170.63	4592.25	38.000	0.869
	Non-Ext.	5	2067.80	9033.03		
Vol 1 (mm^3^)	Ext.	16	-828.38	1134.5	31.000	0.457
	Non-Ext.	5	-1232.20	1969.63		
Vol 2 (mm^3^)	Ext.	16	113.81	1702.21	32.000	0.509
	Non-Ext.	5	532.00	1982.25		
Vol 1+vol 2 (mm^3^)	Ext.	16	-714.56	2415.73	35.000	0.680
	Non-Ext.	5	-700.2	3438.82		
Vol 3 (mm^3^)	Ext.	16	3184.19	2300.77	34.000	0.620
	Non-Ext.	5	1764.6	4670.47		
Vol 4 (mm^3^)	Ext.	16	1701.00	1592.14	31.000	0.457
	Non-Ext.	5	1003.4	1814.12		
SA 0 (mm^2^)	Ext.	16	-22.20	103.11	30.000	0.409
	Non-Ext.	5	12.08	147.47		
SA 1 (mm^2^)	Ext.	16	33.75	140.71	39.000	0.934
	Non-Ext.	5	51.36	136.28		
SA 2 (mm^2^)	Ext.	16	147.94	94.29	23.000	0.160
	Non-Ext.	5	66.23	108.15		
SA 3 (mm^2^)	Ext.	16	39.67	93.87	38.000	0.869
	Non-Ext.	5	60.49	97.98		
MSA (mm^2^)	Ext.	16	98.99	88.42	37.000	0.804
	Non-Ext.	5	62.18	92.05		
AP width 0 (mm)	Ext.	16	0.42	2.64	18.000	0.069
	Non-Ext.	5	3.73	4.47		
TV width 0 (mm)	Ext.	16	-1.37	4.55	30.000	0.409
	Non-Ext.	5	0.24	3.71		
AP width 1 (mm)	Ext.	16	1.89	3.04	35.000	0.680
	Non-Ext.	5	2.32	4.12		
TV width 1 (mm)	Ext.	16	-0.93	5.66	32.000	0.509
	Non-Ext.	5	0.98	5.94		
AP width 2 (mm)	Ext.	16	5.34	3.53	30.000	0.409
	Non-Ext.	5	3.69	4.96		
TV width 2 (mm)	Ext.	16	2.42	4.34	29.000	0.364
	Non-Ext.	5	0.34	5.40		
AP width 3 (mm)	Ext.	16	2.10	3.20	32.000	0.509
	Non-Ext.	5	3.39	3.90		
TV width 3 (mm)	Ext.	16	-0.52	3.05	20.000	0.099
	Non-Ext.	5	1.30	2.40		

## Data Availability

The data used to support the findings of this study are included within the article.
